# Treatment of Stricture of the Urethra by Forcible Rupture

**Published:** 1865-10

**Authors:** 


### CHICAGO MEDICAL EXAMINER.

N. S. DAVIS, M.D., Editor.

VOL. VI. OCTOBER, 1865. NO. 10.

### Original Contributions.

### ARTICLE XXXIII.

### TREATMENT OF STRICTURE OF THE URETHRA BY FORCIBLE RUPTURE.

Before proceeding to speak of the treatment of stricture of the urethra, permit me to call your attention to a few points of the surgical anatomy of this canal, which it is very important for every surgeon to understand, before he should attempt to introduce instruments into it. When the penis is lifted up—but not distended—a catheter has only to pass, at most, $7\frac{1}{2}$ inches, when it will enter the bladder.

The urethra is divided into three portions—the prostatic, membranous, and spongy. The extent of the prostatic portion being from 6 to 10 lines in length; of the membranous, 6 to 8 lines; while that of the spongy portion is subject to the greatest variation, and is the only part of the urethra in which the different positions,—and extension—make a change in the direction of the canal.

When a straight catheter is introduced into the bladder of a dead subject laid out on a table, the instrument describes, in relation to the surface of the table, and the axis of the body, an angle of 45°. When the penis is raised, the angle in the spongy portion is effaced; but the curve in the membranous and prostatic portions remains the same.

The direction of the canal in the quiescent state has been compared to the letter S, which is true, when the bladder and rectum are distended.

When the penis is laid against the abdomen, only one curve is found. The urethra is very far from being straight, but it may be rendered so.

The parietes of the canal are soft and yielding, and capable of being extended, in the male, one inch without doing injury to the parts; while, in the female, the urethra may be so distended that calculus of the size of an almond may be extracted from the bladder through this canal. This passage is very frequently the seat of stricture, in fact, more frequently than all the remaining outlets of the body combined.

In the introduction of an instrument into the bladder, the rule of the veteran surgeon, Dr. Mott, is an excellent one, viz., "Make haste slowly."

Every surgeon must be aware of the difficulties attending the treatment of this form of disease.

Without stopping to speak of the usual modes of treatment recommended by most teachers, and in systematic works of surgery, I propose to offer for your consideration an entirely new method (as far as I know) of treating this formidable disease. It is one that has been adopted and used in the United States Marine Hospital, of this City, for the last four years, also, during my term of service, at the Mercy Hospital—and once in private practice—with abundant success. I refer to the treatment by forcible rupture. The instruments used are these which I hold in my hand.

I claim for this method of treatment the following advantages over the old methods:—

1st. It is more expeditious.

2d. In the hands of judicious operators, more safe.

3d. Is not so apt to be followed by relapses.

Every surgeon is aware of the length of time necessary to treat a stricture, by dilatation, for instance. I have known instances where weeks and even months elapsed before a cure was effected.

The danger attending this form of treatment is considerable, also. The liability to make false passages is great; in fact, that method of treatment can only be conducted by skilful hands.

In the treatment by rupture, a very few days, at the longest, suffice to effect a cure.

2. In the hands of judicious operators it is more safe.

Under this head I will simply state, that out of the large number of cases treated by this method, I have never known any bad symptoms follow. It is in use at the Marine Hospital almost every week, where I have had opportunity to observe its merits.

3. Not so liable to be followed by relapses.

The liability which exists for the return of a stricture after treatment by dilatation, or by caustic, is excessive, and is noticed by all authors. Erichsen says: "There is great tendency to relapse, and for the constriction to return, the stricture rapidly becoming tighter as soon as the introduction of intruments is discontinued."

I have not observed this to follow after treatment by rupture. The cases in which this method of treatment is applicable, are all forms of organic stricture, when the smallest-sized sound can be passed. It is necessary to place the patient under the influence of chloroform during the operation. I might cite cases illustrating this method of treatment, but as I have not any prepared, I will content myself by reporting one that occurred under my care at the Mercy Hospital.

Thomas Ott, American, aged 24, was admitted into the Mercy Hospital, March 1st, 1859, with stricture and spermatorrhœa, which affections he had had for four years. The cause of the stricture, which was situated in the membranous portion of the urethra, was, in all probability, the repeated applications of caustic, for the cure of the spermatorrhœa. He was treated during the month of March by gradual dilatation, but without much apparent benefit. On the first of April, that being the commencement of my term of service, he came under my care. It was with difficulty that a No. 2 sound could be introduced at this time. I continued the introduction of sounds until I could introduce a No. 3, when I introduced the small-sized rupture instrument, and opened it to its full extent; a slight hemorrhage followed the operation, but nothing more. On the 1st of May, I operated with the larger-sized instrument, which was followed by no bad symptoms whatever.

The patient continued in the Hospital until the 15th of the following June, under treatment for his spermatorrhœa, during which time a large-sized sound was passed. There was no obstruction whatever. I have heard from the patient once since. He remains entirely well. Professor Davis observed the treatment of this patient, and its results.

